# Analysis of six novel flavin-containing monooxygenase 3 (*FMO3*) gene variants found in a Japanese population suffering from trimethylaminuria

**DOI:** 10.1016/j.ymgmr.2015.10.013

**Published:** 2015-11-07

**Authors:** Makiko Shimizu, Yumi Origuchi, Marika Ikuma, Nanako Mitsuhashi, Hiroshi Yamazaki

**Affiliations:** Laboratory of Drug Metabolism and Pharmacokinetics, Showa Pharmaceutical University, 3-3165 Higashi-tamagawa Gakuen, Machida, Tokyo 194-8543, Japan

**Keywords:** Flavin-containing monooxygenase 3, Fish odor syndrome, Trimethylamine, Polymorphism, Japanese, Trimethylaminuria

## Abstract

Polymorphic human flavin-containing monooxygenase 3 (FMO3) is associated with the inherited disorder trimethylaminuria. Several FMO3 variants have been observed in a variety of ethnic groups, including a Japanese cohort suffering from trimethylaminuria. The aim of this study was to screen another self-reported Japanese trimethylaminuria cohort for novel FMO3 variants and to investigate these new variants. Subjects with low FMO3 metabolic capacities were identified by measuring the urinary trimethylamine and trimethylamine *N*-oxide concentrationsin171 Japanese volunteers. The *FMO3* genes from these subjects and their family members were then sequenced. Heterozygotes or homozygotes for novel single-nucleotide polymorphisms c.20 T > C p.(Ile7Thr), c.122 G > A p.(Trp41Ter), c.127T > A p.(Phe43Ile), c.488 T > C p.(Leu163Pro), and c.1127G > A p.(Gly376Glu) and a heterozygote for the novel duplication c.850_860dupTTTAACGATGA p.(Glu287AspfsTer17) were identified. In addition, the known (but as yet uncharacterized) single-nucleotide polymorphism c.929 C > T p.(Ser310Leu) was found. Pedigree analysis revealed the p.(Ser310Leu) *FMO3* allele in *cis* configuration with c.929 C > T p.(Glu158Lys). These variant FMO3 proteins recombinantly expressed in *Escherichia coli* membranes exhibited decreased *N*-oxygenation activities toward trimethylamine and benzydamine. Although the allele frequencies of these seven variants were low, the present results suggest that individuals homozygous or heterozygous for any of these novel missense or duplication*FMO3* variants or known nonsense mutations such as p.(Cys197Ter) may possess abnormal activities toward trimethylamine *N*-oxygenation.

## Highlights

• Further screen for flavin-containing monooxygenase 3 variants was performed.
• p.(Glu287AspfsTer17) and 6 variants of FMO3 were identified in Japanese.
• Variant FMO3 proteins showed low trimethylamine *N*-oxygenation.
• These *FMO3* mutations could be causative of trimethylaminuria.

## 1 Introduction

Polymorphic human flavin-containing monooxygenase 3 (FMO3, EC 1.14.13.8) is associated with the inherited disorder trimethylaminuria [1], [2] — the inability to metabolize odorous dietary-derived trimethylamine to its non-odorous *N*-oxide [3], [4]. Rare loss-of-function mutations [1] that cause the disorder have been reported. Decreased or abolished functional activities with respect to trimethylamine *N*-oxygenation, resulting in trimethylaminuria, are caused mainly by single nucleotide polymorphisms of the *FMO3* gene. Such *FMO3* polymorphisms have been reported in the literature [5], [6], [7], [8], [9]. *FMO3* mutations resulting in the amino acid substitutions p.(Glu158Lys), p.(Val257Met), and p.(Glu308Gly) have been reported as common *FMO3* gene variants in the International HapMap project (http://www.hapmap.org) in multiple ethnic populations. Differences in terms of frequency of occurrence of the *FMO3* variants have been recognized in Caucasian and Asian populations [10].

We previously analyzed the function of six novel FMO3 variants in 640 Japanese volunteers with self-reported trimethylaminuria [3]. Currently, Japanese subjects(from different families) with trimethylaminuria are known to possess p.(Cys197Ter), p.(Arg205Cys), and p.(Arg500Ter) *FMO3* alleles with frequencies in the range of 2–4%. Minor FMO3 variants p.(Val58Ile), p.(Pro70Leu), p.(Asn114Ser), p.[(Glu158Lys;Thr201Lys;Glu308Gly)], p.[(Glu158Lys;Gln470Ter)], p.(Ser195Leu), p.[(Val257Met;Met260Val)], p.[(Val257Met;Trp388Ter)], p.(Gly421Val), p.(Ile441Thr), and p.(Thr488Ala) have also been found in Japanese cohorts [3], [4], [8], [10], [11], [12], [13], [14].

In our 2012 report, we screened and investigated 640 Japanese volunteers with self-reported trimethylaminuria [3]; in this current study, we screened and investigated novel FMO3 variants in a further 171 Japanese volunteers with self-reported trimethylaminuria. We report herein six new FMO3 variants with impaired trimethylamine *N*-oxygenation as causal factors for trimethylaminuria in a Japanese population.

## 2 Materials and methods

The basic experimental methods for screening urinary trimethylamine and trimethylamine *N*-oxide concentrations, sequencing the *FMO3* gene from buccal cells obtained from volunteer subjects suffering from self-reported body malodor, and recombinantly expressing FMO3 variant proteins in bacterial membranes were described previously [3], [4]. The current investigation is a follow-up study to our 2012 report [3] with a new group of 171 subjects ranging from 1 to 93 years of age; informed consent was obtained from each subject or parent of the subject. The ethics committees of Showa Pharmaceutical University approved this study.

Genotyping analysis for the novel mutations was carried out by allele-specific polymerase chain reaction (PCR) methods or PCR-restriction fragment length polymorphism (RFLP) methods, as shown Table 1, with DNA amplified using the primers described previously [15]. RFLP digestions were carried out at 37 °C for 2 h using the designated restriction enzymes. The primers for allele-specific PCR methods for p.(Ile7Thr), p.(Phe43Ile), and p.(Glu287AspfsTer17) variants are listed in Table 2. Trimethylamine, trimethylamine *N*-oxide, benzydamine, benzydamine *N*-oxide, and other reagents were from sources described previously [3].

**Table 1:** PCR-RFLP and allele-specific analyses of *FMO3* variants in a Japanese cohort.

Variant	Position with respect to accession number AL021026	Exon	Restriction enzyme for PCR products	Length (bp) of RFLP products, uncut/cut (fragment)
p.(Ile7Thr)	g.5736 T > C	2	Allele specific (-cat/-cac)	186
p.(Trp41Ter)	g.5838 G > A	2	*Sfc*I	519/273 + 246 (mutant)
p.(Phe43Ile)	g.5843 T > A	2	Allele specific (-aat/-aaa)	256
p.(Leu163Pro)	g.21140 T > C	5	*Sau*96I	699/473 + 226 (mutant)
p.(Glu287AspfsTer17)	g.27086-27096dupTTTAACGATGA	7	–	464/475
p.(Ser310Leu)	g.27165 C > T	7	*Eco*T14I	345 + 119/149 + 196 + 119 (mutant)
p.(Gly376Glu)	g.27363 G > A	7	*Eco*T14I	345 + 119/464 (mutant)

**Table 2:** Sequences of allele-specific primers used for detection of p.(Ile7Thr), p.(Phe43Ile), and p.(Glu287AspfsTer17) variants.

Variant		Primer name	Allele-specific primer
p.(Ile7Thr)	Forward	hFMO3 Ile7Thr wild	5′- GGGAAGAAAGTGGCCAT -3′
	Forward	hFMO3 Ile7Thr mutant	5′- GGGAAGAAAGTGGCCAC -3′
	Reverse	hFMO3ex2AS	5′- GATCTATCAAGGGAGAACTGTA -3′
p.(Phe43Ile)	Forward	hFMO3 Phe43Ile wild	5′- CATTGGGGGCCTGTGGAAAT -3′
	Forward	hFMO3 Phe43Ile mutant	5′- CATTGGGGGCCTGTGGAAAA -3′
	Reverse	hFMO3ex2AS	5′- GATCTATCAAGGGAGAACTGTA -3′
p.(Glu287AspfsTer17)	Forward	hFMO3ex7S	5′- ACAAGAGGGAAATATTACACTTCC -3′
	Reverse	hFMO3-E287Dfs-R1	5′- AATGCTTGCTGGGAGCTC -3′

## 3 Results

### 3.1 DNA analysis of probands 1–8

**Fig. 1:**
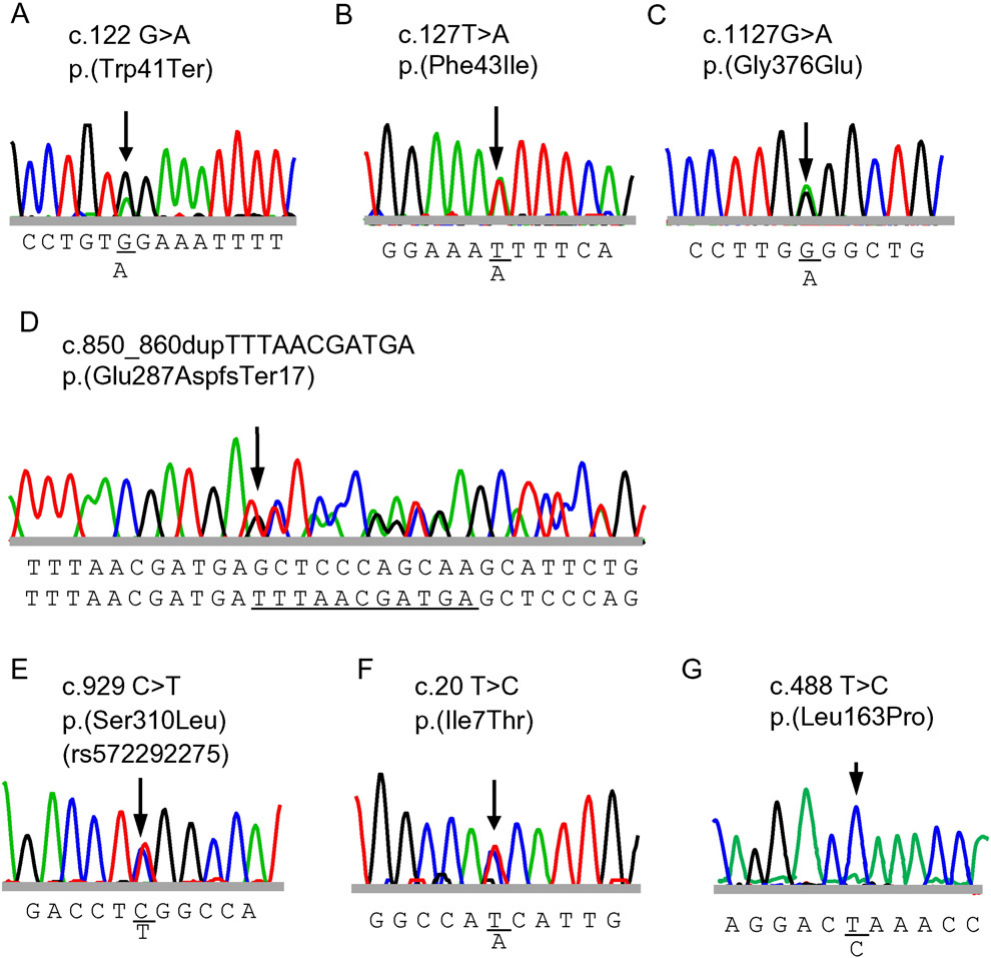
Nucleotide sequences of variant *FMO3*. Both strands were sequenced. The sequences are shown only for sense strands of genomic DNA from probands 1 (A), 2 and 3 (B), 2 and 4 (C), 5 (D), 6 (E), 7 (F), and 8 (G). The sequence of the complete human *FMO3* gene described in GenBank (Accession Number AL021026) was used as the reference.

**Fig. 2:**
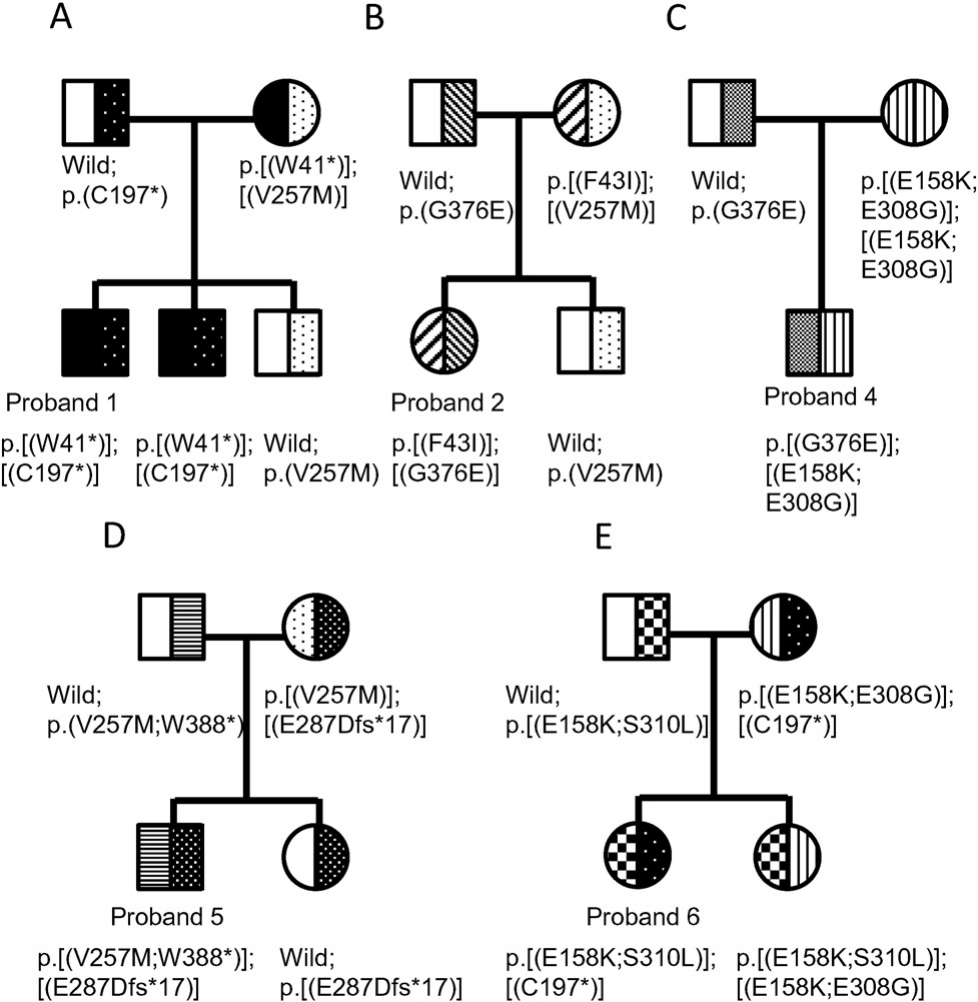
Pedigree analysis for the presence of novel *FMO3* variants in probands 1 (A), 2 (B), 4 (C), 5 (D), and 6 (E).

FMO3 metabolic capacity (% of total trimethylamine excreted as trimethylamine *N*-oxide) was determined in volunteers with self-reported trimethylaminuria. The frequency of subjects with less than 40% FMO3 metabolic capacity (severe trimethylaminuria) was 11% (20 of 171 subjects) in a Japanese population with self-reported trimethylaminuria. We focused on eight of the participants who had low to moderate metabolic capacities for trimethylamine *N*-oxygenation (Table 3). In proband 1, who had 29% FMO3 metabolic capacity, a single-nucleotide polymorphism in exon 2 of *FMO3* at c.122 G > A was observed that resulted in p.(Trp41Ter) FMO3 (Fig. 1A). Proband 1 was heterozygous for this novel *FMO3* variant. Pedigree analysis revealed this *FMO3* p.(Trp41Ter) allele to be in *trans* configuration with p.(Cys197Ter) (Fig. 2A) [12]. One of the brothers of proband 1, who had 19% FMO3 metabolic capacity, had the same genotype as proband 1 (Fig. 2A). To test for the p.(Trp41Ter) *FMO3* gene variant, a simple PCR-RFLP method was developed. As shown in Table 1, the PCR product from the mutant G > A allele at codon 41 could be digested by *Sfc*I into 273- and 246-bp fragments. A more extensive analysis of DNA revealed that the mother of proband 1 was heterozygous for p.(Trp41Ter) (Fig. 1A). Urinary trimethylamine *N*-oxygenation analysis of both parents and another brother of proband 1 showed that their FMO3 metabolic capacity was greater than 90%, in contrast to the < 30%metabolic capacity of proband 1and his brother.

**Table 3:** *In vivo* FMO3 metabolic capacity of probands 1–8 from urine tests.

Proband	Age/gender (years)	Genotype	FMO3 metabolic capacity, %
1	12, M	p.[(Cys197Ter)];[(Trp41Ter)]	29
2	6, F	p.[(Phe43Ile)];[(Gly376Glu)]	31
3	42, M	p.[(Phe43Ile)];[(Cys197Ter)]	19
4	5, M	p.[(Gly376Glu)];[(Glu158Lys;Glu308Gly)]	90
5	4, M	p.[(Val257Met;Trp388Ter)];[(Glu287AspfsTer17)]	24
6	7, F	p.[(Glu158Lys;Ser310Leu)];[(Cys197Ter)]	15
7	21, F	p.[(Arg205Cys)];[(Ile7Thr)]	49
8	37, M	p.[(Leu163Pro)];[(Leu163Pro)]	32

In proband 2, who possessed 31% FMO3 metabolic capacity, we found the novel c.127T > A *FMO3* variant p.(Phe43Ile) (Fig. 1B) and the novel variant c.1127G > A *FMO3* p.(Gly376Glu) (Fig. 1C) (Table 3). DNA sequencing of *FMO3* in samples from proband 2's family (Fig. 2B) revealed that proband 2 and her mother were heterozygous for p.(Phe43Ile) allele and proband 2 and her father were heterozygous for the p.(Gly376Glu) allele. As shown in Table 1, allele-specific PCR and PCR-RFLP methods were developed for p.(Phe43Ile) and p.(Gly376Glu), respectively. These methods could successfully identify the respective *FMO3* variants. The PCR product from the ancestral allele at codon 376 is digested by *Eco*T14I into 345- and 119-bp fragments, whereas the variant allele is not digested. In probands 3 and 4, we found p.(Phe43Ile) and p.(Gly376Glu) alleles, respectively (Fig. 2C). Probands 3 and 4 had FMO3 metabolic capacities of 19% and 90%, respectively (Table 3). These individuals were also heterozygous for the known variants p.(Cys197Ter) and p.[(Glu158Lys;Glu308Gly)] [8], respectively (Table 3).

We found a duplication (TTTAACGATGA) polymorphism at the c.850_860 position of *FMO3* variant p.(Glu287AspfsTer17) (Fig. 1D) in proband 5, who possessed 24% FMO3 metabolic capacity (Table 3). The sequence of this duplication was identical to c.850–860 in exon 7 of ancestral type *FMO3*. Pedigree analysis disclosed that the p.(Glu287AspfsTer17) allele was in the *trans* configuration with p.[(Val257Met;Trp388Ter)] (Fig. 2D). The PCR product lengths from this duplication variant were longer than those of the ancestral allele using the primers shown in Table 2. The mother and sister of proband 5 had this duplication variant, as reconfirmed by this new method.

Another new *FMO3* variant, c.929C > T p.(Ser310Leu) (Fig. 1E), was found inproband6, who possessed 15% FMO3 metabolic capacity (Table 3). Pedigree analysis revealed this p.(Ser310Leu) *FMO3* allele to be in the *cis* configuration with p.(Glu158Lys) (Fig. 2E). On DNA sequencing the *FMO3* genes from this family, we found that proband 6 and her father and sister were heterozygous for the p.[(Glu158Lys;Ser310Leu)] allele (Fig. 2E). As shown in Table 1, a PCR-RFLP method was developed for the p.(Ser310Leu) allele using *Eco*T14I. This method was the same as that for the p.(Gly376Glu) variant. The PCR product from the mutant allele at codon 310 was digested by *Eco*T14I into 149-, 146-, and 119-bp fragments. Urinary analysis of both parents and the sister of proband 6 showed that their FMO3 metabolic capacities were greater than 80%, in contrast to the 15% metabolic capacity of proband 6. We found other novel variants c.20T > C p.(Ile7Thr) (Fig. 1F) in proband 7 and c.488T > C p.(Leu163Pro) (Fig. 1G) in proband 8. Proband 7, who was heterozygous for the above new variant and known mutation p.(Arg205Cys), had 49% FMO3 metabolic capacity (Table 3). Proband 8 was homozygous for the above novel mutation andhad32% *in vivo* FMO3 metabolic capacities (Table 3). The simple allele-specific PCR or PCR-RFLP methods shown in Table 1 were developed and were able to identify these seven *FMO3* variants.

The allele frequencies of p.(Ile7Thr), p.(Trp41Ter), p.(Phe43Ile), p.(Leu163Pro), p.(Glu287AspfsTer17), p.(Ser310Leu), and p.(Gly376Glu) *FMO3* alleles were 0.3% (1 of 342 alleles), 0.3%, (1 of 342 alleles), 0.6% (2 of 342 alleles), 0.6% (2 of 342 alleles), 0.3% (1 of 342 alleles), 0.3% (1 of 342 alleles), and 0.6% (2 of 342 alleles), respectively, in the current Japanese cohort (excluding family members). Although the existence of p.(Trp41Ter), p.(Glu287AspfsTer17), and p.(Ser310Leu) *FMO3* alleles was confirmed in the family of probands 1, 5, and 6, the apparent allele frequencies given above were calculated among unrelated subjects.

### 3.2 Enzyme activities of recombinant FMO3 variants

The kinetic parameters for the functional activities of recombinantly expressed variant FMO3 proteins with respect to trimethylamine and benzydamine *N*-oxygenation were determined by nonlinear regression analysis; these parameters were compared with those of wild type FMO3 expressed in bacterial membranes (Table 4). The apparent *K*_m_ values of the FMO3 variants, except for p.(Ile7Thr) and p.(Gly376Glu), were approximately the same as that for wild type FMO3. However, the apparent *K*_cat_ and *K*_cat_/*K*_m_ values of the FMO3 variants were approximately in the range one-half to one-twentieth of that of the wild-type enzyme (Table 4). FMO3 variants p.(Ile7Thr), p.[(Glu158Lys;Ser310Leu)], and p.(Gly376Glu) had less than10% of wild-type trimethylamine *N*-oxygenation activity.

**Table 4:** Functional activities of wild-type and variant FMO3recombinantly expressed in *E. coli*.

Variant FMO3	*N*-oxygenation					
	Trimethylamine			Benzydamine		
	*K*_m_, μM	*K*_cat_, min^− 1^	*K*_cat_/*K*_m_	*K*_m_, μM	*K*_cat_, min^− 1^	*K*_cat_/*K*_m_
Wild-type	70 ± 9	50 ± 2	0.7 (100)	58 ± 5	197 ± 4	3.4 (100)
7Thr	429 ± 215	2.4 ± 0.6	0.006 (0.9)	50 ± 6	0.73 ± 0.02	0.02(0.6)
43Ile	46 ± 9	6.5 ± 0.24	0.14 (20)	49 ± 5	23 ± 0.5	0.47 (14)
163Pro	60 ± 15	19 ± 1	0.31 (44)	59 ± 9	90 ± 4	1.5 (44)
158Lys;310Leu	83 ± 20	1.6 ± 0.1	0.02 (3)	61 ± 4	59 ± 1	0.97 (29)
376Glu	33 ± 18	1 ± 0.1	0.03 (4)	22 ± 7	0.27 ± 0.02	0.01 (0.3)

The substrates (0–500 μM trimethylamine and 0–1000 μM benzydamine) were incubated with recombinant FMO3 (50 and 5 pmol Eq for trimethylamine and benzydamine oxygenations) at 37 °C for 30 and 10 min, respectively, in the presence of an NADPH-generating system. Kinetic parameters were calculated from a fitted curve by nonlinear regression (mean ± SE). Values in parentheses are percentages of the wild type value.

## 4 Discussion

A human FMO3 database [16] has been established (http://databases.lovd.nl/shared/genes/FMO3) by scientists in the United Kingdom and contains known causative FMO3 variants of trimethylaminuria [10], [17]. In the current study, six novel FMO3 variants with impaired trimethylamine and benzydamine *N*-oxygenation capacity were discovered in a Japanese population with self-reported trimethylaminuria; these variants are likely causative of trimethylaminuria (Fig. 1). In addition to these six novel FMO3 variants, FMO3 p.(Ser310Leu) (rs572292275) was also found in our cohort. This FMO3 variant has been reported in a Portuguese population [18], but no functional analysis has yet been carried out. In the current study, p.[(Glu158Lys;Ser310Leu)] FMO3 variant was found in a familial analysis; this variant exhibited extremely impaired trimethylamine and benzydamine *N*-oxygenation capacity (Table 4). An FMO3 protein truncated at position 303 resulted from the 10-base duplication in variant p.(Glu287AspfsTer17). The functional importance of the *C*-terminus of human FMO3 was clearly indicated by our previous findings that recombinant p.(Arg500Ter) FMO3 expressed in bacterial membranes showed no detectable functional activity toward trimethylamine. These results suggest that individuals harboring the nonsense *FMO3* mutations p.(Trp41Ter), p.(Cys197Ter), p.[(Val257Met;Trp388Ter)], and p.(Glu287AspfsTer17) will likely exhibit abnormally low levels of trimethylamine *N*-oxygenation.

The eight probands in this study were heterozygotes or homozygotes for novel or known FMO3 variants (Fig. 1) and did not efficiently *N*-oxygenate dietary-derived trimethylamine to its *N*-oxide (Table 3). The impaired levels of trimethylamine *N*-oxygenation evident in the *in vivo* phenotype (Table 3) were most likely caused by the combination of these known and/or novel *FMO3* variants (Table 4).

In conclusion, subjects carrying heterozygous/homozygous combinations of any of the nonsense or missense mutated *FMO3* alleles found in this study, together with those previously reported, probably possess FMO3 with reduced trimethylamine *N*-oxygenation efficiency and thus may suffer from severe or mild trimethylaminuria.
